# The expression of Toll-like receptor 5 in preterm histologic chorioamnionitis

**DOI:** 10.3164/jcbn.16-103

**Published:** 2017-10-11

**Authors:** Hiroaki Moroi, Tomomi Kotani, Rika Miki, Hiroyuki Tsuda, Masako Mizuno, Yumiko Ito, Takafumi Ushida, Kenji Imai, Tomoko Nakano, Hua Li, Seiji Sumigama, Eiko Yamamoto, Akira Iwase, Fumitaka Kikkawa

**Affiliations:** 1 Department of Obstetrics and Gynecology, Nagoya Graduate University School of Medicine, 65 Tsurumai-cho, Showa-ku, Nagoya 466-8550, Japan; 2 Department of Obstetrics and Gynecology, Handa City Hospital, 2-29 Toyo-cho, Handa-shi, Aichi 475-8559, Japan; 3 Laboratory of Bell Research Centre–Department of Obstetrics and Gynaecology Collaborative Research, Nagoya Graduate University School of Medicine, 65 Tsurumai-cho, Showa-ku, Nagoya 466-8550, Japan; 4 Department of Obstetrics and Gynecology, Japanese Red Cross Nagoya Daiichi Hospital, 3-35 Michishita-cho, Nakamura-ku, Nagoya 453-8511, Japan; 5 Department of Neurology, Yanbian University Hospital, 1327 JuZi Street, Yanji City, JiLin Province 133000, China; 6 Office of international Affairs, Nagoya Graduate University School of Medicine, 65 Tsurumai-cho, Showa-ku, Nagoya 466-8550, Japan; 7 Department of Healthcare Administration, Nagoya Graduate University School of Medicine, 65 Tsurumai-cho, Showa-ku, Nagoya 466-8550, Japan

**Keywords:** amnion, chorion, IL-6, IL-8

## Abstract

Spontaneous preterm birth is often caused by chorioamnionitis. Toll-like receptors (TLRs) have a role in the response of the innate immune system. The role of TLR5 in chorioamnionitis remains unclear: however, TLR5 was reported to have a significantly stronger effect on the induction of interleukin (IL)-6 when compared with other TLRs in amniotic epithelial cells. The aim of this study was to investigate TLR5 expression in placentas with preterm histologic chorioamnionitis (HCA). The expression levels of TLR5 were evaluated in the amnions, chorions, deciduae and villi with and without HCA using immunohistochemistry. The co-localization of IL-6 or IL-8 with TLR5 was examined by immunofluorescence. The production of IL-6 was examined in primary tissue cultured fetal membranes treated with and without the TLR5 agonist. The protein expression of TLR5 was significantly increased in amnions with HCA (*p*<0.05) and showed a trend toward an increase in chorions with HCA, whereas no significant difference was detected in the villi and decidua. TLR5 co-localized with IL-6 and IL-8 in amnions and chorions. IL-6 showed a significant increase (*p*<0.05) with the TLR5 agonist. These results suggest that TLR5 plays a role in the pathogenesis of preterm HCA and IL-6 production.

## Introduction

An estimated 14.9 million babies were born preterm in 2010, which represents 11.1% of all livebirths worldwide with a range of approximately 5% to 18%.^(1)^ The preterm birth rate is increasing worldwide, and it is the leading cause of death in children younger than 5 years of age.^(1)^ Ascending microorganism infections are one of main causes of spontaneous preterm birth. The invasion of microorganisms into the amniotic cavity activates a host immune response that causes inflammation in fetal membranes, chorioamnionitis. Intrauterine inflammation can also cause uterine contractions and an adverse effect on the fetus.^(2)^ Chorioamnionitis is associated with adverse outcomes in preterm neonates.^(3)^

Toll-like receptors (TLRs) are important for pathogen recognition and defense against invading microorganisms. The role of TLRs in chorioamnionitis remains unclear. TLR1 and TLR2 expressions were significantly increased in fetal membranes with preterm histologic chorioamnionitis (HCA), whereas TLR4 and TLR6 expressions were unaffected.^(4,5)^ A previous study determined that TLR4 expression was increased in Hofbauer cells in preterm chorioamnionitis placentas.^(6)^ TLR5 recognizes bacterial flagellin, a component of bacterial flagella, in both Gram-positive and Gram-negative bacteria.^(7)^ TLR5 was recently reported to have a most significant effect on IL-6 and IL-8 production in the amniotic epithelial cells among the members of TLR family.^(8)^ Elevated IL-6 and IL-8 concentrations in amniotic fluid are sensitive tests for the prospective diagnosis of chorioamnionitis.^(9–11)^ In addition, it was reported that TLR5 mRNA expression among the members of TLR family is only increased in the placenta with chorioamnionitis, although the samples were collected from mixed term and preterm subjects.^(12)^ However, these findings are partial evidence, thus, additional work is needed to confirm if TLR5 is involved in chorioamnionitis pathology.

In the present study, we examined the protein expression and localization of TLR5 in placentas with and without preterm HCA. Moreover, we investigated the effect of TLR5 activation via flagellin (TLR5 agonist) on IL-6 and IL-8 production in fetal membranes.

## Materials and Methods

### Immunostaining

The procedure was approved by the ethics committee of the Nagoya University School of Medicine (approval number: 0032). Samples were obtained from 15 placentas from preterm birth, which was fixed in formalin, and embedded in paraffin. Cases of non-singleton, placenta previa, preeclampsia or chromosomal abnormalities of fetus were excluded from this study. Seven samples showed histologically diagnosed HCA based on Blanc’s criteria. We also included 8 preterm gestational age-matched control samples from subjects were terminated pregnancy by therapy due to maternal disease except obstetrical complications. The clinical characteristics of the patients are summarized in Table 1.

Immunostaining and immunofluorescence were performed as previously described.^(13,14)^ Briefly, the samples were fixed in formalin and embedded in paraffin. For immunostaining of TLR5, sections retrieved epitope in 10 mM citrate buffer (pH 6.0) at 95°C by microwave for 20 min were incubated with rabbit anti-TLR5 Ab (ab62460, Abcam, Tokyo, Japan: dilution of 1:150) at 4°C overnight. Detection was performed with the avidin–biotin immunoperoxidase technique, using the Histofine SAB-PO kit (Nichirei, Tokyo, Japan), according to the manufacturer’s protocol. Images were obtained using a BZ-9000 microscope (Keyence, Osaka, Japan). The intensity of TLR5 immunoreactivity was semiquantitatively evaluated on a scale of 0 to 3 (0: none, 1: weak, 2: moderate, 3: strong) by two examiners dependently.

For double-labeled immunofluorescence, the sections were incubated with rabbit anti-TLR5Ab (ab62460, Abcam: dilution of 1:150) and mouse anti-IL-6Ab (ab9324, Abcam: dilution of 1:150) or mouse anti-IL-8Ab (ab18672, Abcam: dilution of 1:150) at 4°C overnight. Alexa Fluor 488-labeled goat anti-rabbit antibody (A11034; Thermo Fisher Scientific, Waltham, MA) and Alexa Fluor 594-labeled goat anti-mouse antibody (A11032; Thermo Fisher Scientific) were used as a secondary antibody, respectively. Nuclei were labeled with 4',6-diamidino-2-phenylindole dihydrochloride (DAPI, Roche Basel, Switzerland) , for staining of nucleic acid. Fluorescence mounting medium (Dako North America, Carpinteria, CA) was applied and images were obtained using IX71 microscope with DP72 camera and cellSence software (OLYMPUS, Tokyo, Japan). For the negative controls, primary antibody was replaced by a nonspecific immunoglobulin G (IgG) at the same dilution.

### Quantification of IL-6 and IL-8 in primary cultured fetal membrane

The procedure was approved by the ethics committee of the Nagoya University School of Medicine (approval number: 414) and informed consent was obtained from each patient. The chorioamniotic membranes (i.e., fetal membranes, *n* = 10) were aseptically obtained via elective Caesarean section before the onset of labor for breech presentation or previous Caesarean section. The intact tissues pieces were cultured in DMEM/F12 (11039-021; Thermo Fisher Scientific) containing 10% fetal calf serum (FCS) (Biological Industries, Haemek, Israel), 100 U/ml penicillin G (Meiji Seika Pharma Co., Ltd., Tokyo, Japan), 100 µg/ml streptomycin (Meiji Seika Pharma Co., Ltd.) and 2.5 µg/ml amphotericin B (Sigma Aldrich, St. Louis, MO) at 37°C under a 5% CO_2_ atmosphere for 8 h. The tissues were incubated in DMEM/F12 at 37°C under a 5% CO_2_ atmosphere, with or without the treatment of agents after 1 h of starvation. The concentration of each ligand was determined as a previous report: 100 ng/ml of Pam3Cys-Ser-(Lys)4 (Novus Biologicals, Littleton, CO); 50 µg/ml of poly(I:C) (Novus Biologicals); 1 µg/ml of LPS (Sigma-Aldrich Japan, Tokyo, Japan); 100 ng/ml of flagellin (Novus Biologicals); and 100 ng/ml of MALP2 (Novus Biologicals).^(8)^ TNF-α at a concentration of 10 ng/ml (Novus Biologicals) and 10 ng/ml of IL-1β (Novus Biologicals) were also used.

For the stimulation experiments, the fetal membranes were treated with the agents above mentioned for 24 h, and the culture supernatants were collected and assayed. The productions of IL-6 and IL-8 were normalized to the weight of each tissue sample. The supernatants were tested using a Quantikine ELISA Human IL-6 Immunoassay Kit (R&D Systems Inc., Minneapolis, MN) and IL-8 Immunoassay Kit (R&D Systems Inc.) according to the manufacturer’s instructions.

### Statistical analysis

The statistical analyses were performed using the SPSS 24 software package (SPSS Inc., Chicago, IL). For comparisons between two groups, Student’s *t* test or Mann-Whitney’s *U* test was used. Appropriate method was selected whether samples were normally distributed. Differences between the groups were considered to be statistically significant at *p*<0.05.

## Results

### Characteristics

The clinical characteristics of women with and without preterm HCA were shown in Table 1. The maternal age in the control group was older than that in the preterm HCA group; however, this difference was not statically significant. No significant difference between two groups was also detected in the other characteristics.

### TLR5 expression in the placenta

**Fig. 1:**
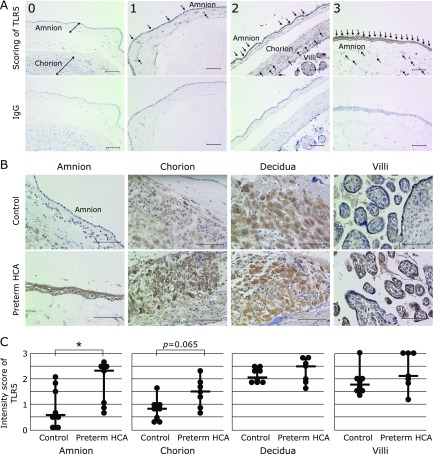
The TLR5 staining of preterm placentas with histologic chorioamnionitis (preterm HCA), compared with them without HCA (control). (A) Representative images for the intensity of TLR5 immunoreactivity (upper panel) on a scale of 0 to 3 (0: none, 1: weak, 2: moderate, 3: strong). Each negative control for the images is shown in the lower panel. Scale bar = 100 µm. (B) Representative images of the expression of TLR5 in control placentas (upper panels) and placentas with HCA (lower panels). Scale bar = 100 µm. (C) The data of comparison of TLR5 expression at different tissues (amnion, chorion, decidua and villi) between with and without HCA are shown as the mean ± SD. *p<0.05 by Mann-Whitney’s *U* test.

 Immunohistochemical analysis against TLR5 was performed to between the placentas with preterm HCA and control placentas. TLR5 expression was significantly stronger in amnions with preterm HCA, when compared with control samples (Fig. 1B and C, *p*<0.05). It was also higher in chorions with preterm HCA, but it could not reach to a statistical significance (Fig. 1B and C, *p* = 0.065). No significant difference was detected in the deciduae and villi between the two groups (Fig. 1B and C).

### TLR5 activation induces IL-6 secretion from the fetal membranes

**Fig. 2:**
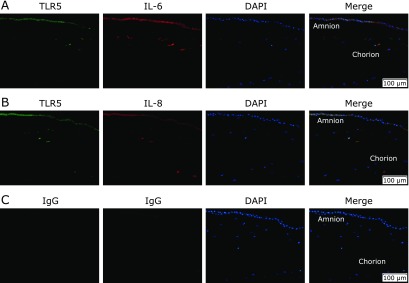
Representative images for the expressions of TLR5 (green), IL-6 (red, A), IL-8 (red, B) and co-expressions of TLR5 and IL-6 (merge, A) or IL-8 (merge, B) in amniochorions with HCA. Negative controls for TLR5, IL-6 and IL-8 (C). Nuclei were labeled with DAPI (blue). Scale bar = 100 µm.

To assess the interaction between high TLR5 expression in fetal membrane and IL-6 or IL-8 production, we performed double immunostaining. IL-6 (Fig. 2A) and IL-8 (Fig. 2B) co-localized with TLR5 in amnions and chorions with HCA.

**Fig. 3:**
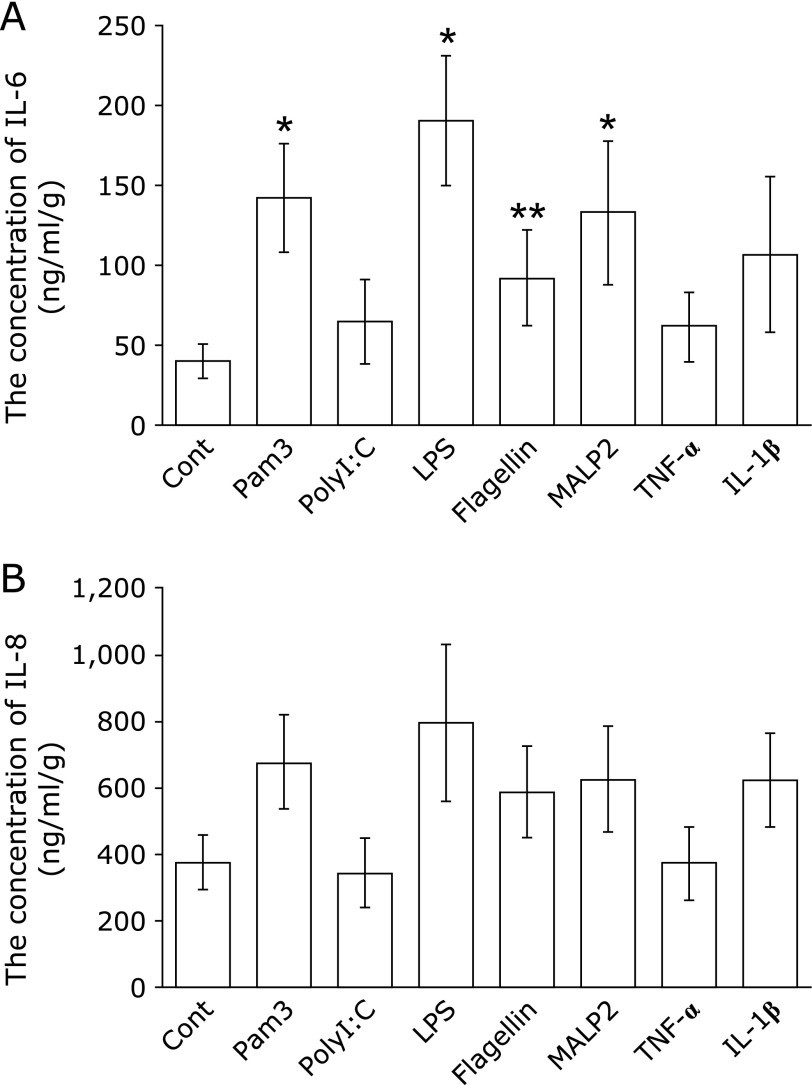
The effects of TLR1–6 ligands and pro-inflammatory cytokines on IL-6 (A) and IL-8 (B) production in fetal membranes (*n* = 10). The data are shown as the mean ± SD. *p<0.01, **p<0.05 by Mann-Whitney’s *U* test.

In primary tissue cultured fetal membranes, TLRs1–6 ligands were evaluated for their potential to induce of IL-6 and IL-8. IL-6 showed a significant increase after treatment with Pam3Cys-Ser-(Lys)4 (ligand of TLR1 and 2), LPS (ligand of TLR4), flagellin (ligand of TLR5) and MALP2 (ligand of TLR2 and 6), when compared with the control (Fig. 3A, *p*<0.01, *p*<0.01, *p*<0.05 and *p*<0.01, respectively). IL-6 production was not affected by the treatment of poly(I:C) (ligand of TLR3), TNF-α or IL-1β, when compared with that observed in control (Fig. 3A). The concentration of IL-8 showed a non-significant increase after treatment with Pam3 Cys-Ser-(Lys)4, LPS, flagellin and MALP2 when compared with the control (Fig. 3B).

## Discussion

This is the first study to demonstrate that TLR5 protein expression was significantly increased in the amnion and showed a trend toward an increase in the chorion with preterm HCA. TLR5 coexisted with IL-6 and IL-8 in the amnion and chorion of preterm HCA. It is well established that IL-6 and IL-8 are increased in response to HCA or intrauterine inflammation. Flagellin, a ligand of TLR5, significantly increased IL-6 production in the fetal membranes. A non-significant increase in IL-8 production was detected. TLR5 was also observed in the villi and deciduae from preterm HCA.

Recently, it has been reported that TLR5 signaling is associated with pathogenesis of inflammatory bowel disease,^(15)^ rheumatoid arthritis,^(16)^ cardiac fibrosis,^(17)^ and cystic fibrosis lung disease.^(18)^ These reports suggested that over-activation or increased expression of TLR5 exacerbates the inflammatory response; thus TLR5 may be a therapeutic target for these diseases. The increased expression of TLR5 and subsequent induction of IL-6 in the amnion and chorion is thought to be triggered for protection of ascending infections; however, these changes may be related to the aggravation of inflammation due to HCA and adverse outcomes. In our study, we found no relationship between vaginal bacteriosis and TLR5 expression (data not shown). However, TLR5 was also observed in the deciduae and villi from preterm placenta without HCA. These findings suggest that TLR5 signaling might play a physiological role in maternal-fetal interface.

Knowledge on the role of TLR5 in preterm birth has been limited. It was recently reported that TLR5 is expressed in human amniotic epithelial cells and TLR5 activation induces the largest increase in IL-6 production among TLRs1–10.^(8)^ In a study of term placenta, only TLR2 and TLR5 were elevated by labor onset, and IL-6 production in placental explants also showed the largest response to TLR5 agonists.^(19)^ Those results suggest that TLR5 influences IL-6 production at labor onset in the human placenta. Allelic variations in the *TLR5* gene were reported to be associated with sepsis in preterm infants.^(20)^ An association with preterm neonates without sepsis was also found, suggesting that TLR5 may play a role in preterm birth via inflammatory pathways. A most recent study reported that TLR5 mRNA expression was increased in placenta with chorioamnionitis.^(12)^ Moreover, the present study demonstrated that TLR5 protein expression was increased and showed a trend toward an increase, in the amnion and chorion respectively, i.e., the fetal membrane, and not affected in the decidua and villi with preterm HCA. The stimulation by TLR5 agonist also caused IL-6 production in the fetal membrane, as well as TLR1/2, TLR4, and TLR2/6 agonists, which is consistent with the previous reports.^(8,21,22)^ These finding suggests that TLR5 is associated with the IL-6 production in the fetal membranes with preterm HCA. IL-6 produced from the fetal membrane was secreted into the amniotic cavity. Thus, TLR5 would be involved in the increased level of amniotic fluid IL-6. It is well known that a higher level of IL-6 in the amniotic fluid is related with the invasion of microorganisms into the amniotic cavity, preterm birth and HCA.^(9,10)^ In addition, it is also associated with adverse outcome of neonates.^(23,24)^ TLR5 might play a key role on preterm birth, and the prognosis of preterm neonates, although further research is needed.

In this study, a trend toward an increase of IL-8 by flagellin was demonstrated, although it could not be reached to a significant increase. The previous study reported that flagellin, the TLR5 agonist, induced IL-8 in amniotic epithelial cells.^(8)^ However, in this study, the production in the fetal membrane including amniotic epithelial cells, mesenchymal cells, and chorion was examined, because of the evaluation of the total production. Thus, the difference might be dependent on different tissues. IL-8 has been reported to be significantly increased in amnion with preterm HCA, but not in chorion with preterm HCA.^(5)^ HCA is defined as the infiltration of neutrophils into the chorioamniotic membranes, which is mainly recruited by IL-8, as a chemokine. Moreover, the gradient of its concentration in the chorioamniotic membranes is responsible for diffuse amniotropic infiltration of neutrophils into the membranes.^(25)^ The different responsiveness to flagellin between amnion and chorion might be related with this gradient. The concentration of IL-8 in amniotic fluid complicated with HCA is also well known to be increased, and it would be mainly resulted from the induction of IL-8 in amniotic epithelium. There is one paper reported that IL-8 in the chorioamniotic membranes is significantly increased by flagellin,^(22)^ which might be caused from the high dose of flagellin, in comparison with that in the present study.

Our study has several limitations. Indeed, the study population was too small to evaluate the relationship between TLR5 levels in the amnion and the prognosis of newborns. In conclusion, the present study suggests that the expression of TLR5 is associated with preterm HCA and IL-6 production in fetal membranes. This finding enhances our pathological understanding of HCA and the function of TLR5. Further studies are needed to clarify the role of TLR5 in preterm HCA.

## Figures and Tables

**Table 1:** Clinical characteristics of the patients

	Control	Preterm HCA	*p* value
Number of patients	8	7	
Maternal age (year)	34.00 ± 2.73	31.57 ± 2.23	0.084
Gravida	1.25 ± 1.49	1.57 ± 1.62	0.695
Parity	0.75 ± 0.89	0.57 ± 0.79	0.689
Maternal BMI (kg/m^2^)	23.20 ± 3.79	26.17 ± 8.19	0.373
Gestational age (week)	29.71 ± 2.75	30.31 ± 4.78	0.77
Birth weight (g)	1452 ± 400	1524 ± 142	0.81
Blanc’s criteria			
I		3	
II		0	
III		4	

Data are presented as the means ± SD. *P* value by Student’s *t* test. Histological diagnosis of chorioamnionitis was determined on Blanc’s Criteria.

## Abbreviations

HCA: histologic chorioamnionitis
IL: interleukin
TLR: toll-like receptor
